# A Flexible Microarray Data Simulation Model

**DOI:** 10.3390/microarrays2020115

**Published:** 2013-04-17

**Authors:** Doulaye Dembélé

**Affiliations:** Microarray Platform, IGBMC, CNRS-INSERM-UdS, 1 rue Laurent Fries, Parc d’Innovation, 67400 Illkirch, France; E-Mail: doulaye@igbmc.fr; Tel.: +33-388-653-528

**Keywords:** microarray data, simulation model, R code

## Abstract

Microarray technology allows monitoring of gene expression profiling at the genome level. This is useful in order to search for genes involved in a disease. The performances of the methods used to select interesting genes are most often judged after other analyzes (qPCR validation, search in databases...), which are also subject to error. A good evaluation of gene selection methods is possible with data whose characteristics are known, that is to say, synthetic data. We propose a model to simulate microarray data with similar characteristics to the data commonly produced by current platforms. The parameters used in this model are described to allow the user to generate data with varying characteristics. In order to show the flexibility of the proposed model, a commented example is given and illustrated. An R package is available for immediate use.

## 1. Introduction

Microarray is now a mature technology in the field of molecular biology for monitoring of gene expression profiling at the genome level. Using two-color microarray technology, the biological activities of two samples are compared on an array on which thousands of specific deoxyribonucleic (DNA) sequences are printed or synthesized *in situ*. Two fluorescent dyes (generally red and green) are used for labeling of the samples for hybridization. Then, after washing, scanning and quantification of images corresponding to the red and green fluorescence of the array, numerical values, or intensities are associated with each probe (gene). These values are normalized to correct for undesirable technical variations [1,2]. Using one-color microarray technology, each sample is hybridized on one array. One fluorescence color is used, and expression intensities are obtained for genes on the arrays used. These values should also be normalized [3]. For a given gene, a ratio of values from two samples is used as a measurement of its expression change. Throughout this paper, we use logarithm two scale to transform the values. Hence, we use $log_{2}$ intensities and $log_{2}$ ratios for sample intensities and their ratios. In general, $log_{2}$ ratios are used for two-color microarray data, while $log_{2}$ intensities are used for one-color microarray data.

In a screening study, the goal is to select genes with differential expression from data whose characteristics are unknown. To study the performance of gene selection methods, we often use synthetic data, which can also serve in developing new methods. To properly play their role, synthetic data must resemble as closely as possible the real data they represent. This is achieved by simulating the physical phenomena through a model. The parameters of this model are approximations of the physical laws that govern the observed phenomenon, and a good knowledge of the physical laws is therefore required to obtain a good model. A complex model that takes into account several components of a phenomenon may become less flexible and may be difficult to modify in order to include new factors. In contrast, a simple model can be effective and easy to modify to take into account an unexpected situation.

One way to generate synthetic data is to use real microarray values as seeds [4,5,6,7]. In [4], data were generated using a normal distribution and a real microarray dataset. This dataset was used to estimate some hyperparameters, and the percentage of the differentially expressed (DE) genes was fixed; see details in [4]. The simulation data obtained in [4] may be useful for statistical methods, but they differ from observed data, since the $log_{2}$-intensities obtained vary between the unrealistic bounds 16 and 30. In [5], two samples are selected from the control and the test samples. The measurements associated with their genes are modified (exchange of values between the two samples…) in order to obtain two statistically undistinguishable samples. Finally, a given number of down- and up-regulated genes is used in this dataset. This procedure necessitates a real microarray dataset, which cannot be available. The procedure proposed in [6] is a modular system, including all steps of the microarray technology. This includes slide layout, hybridization, scanning and image processing. Many models already available in the literature are used with new ones in [6]. The system of [6] results in data as close as possible to real biological data, but is quite complex, and the large number of its parameter settings may discourage its use. In [7], two simulation methods are used. In the first, the number of DE genes and the number of levels of changes for these genes are fixed. Then, for each gene, a mean and a standard deviation are drawn from a uniform distribution. Finally, these data (mean and standard deviation) are used as normal distribution parameters to get expression values for the genes. In the second simulation method, the mean and standard deviation of test ($\mu_{t}$, $\sigma_{t}$) and control ($\mu_{c},\sigma_{c}$) samples are estimated from observed real data and are used as normal distribution parameters to get expression values for all genes. There is no flexibility in the number of the DE genes of the method in [7]. A hierarchical model is used in [8,9], where the variance of each gene is simulated using two parameter settings and the $\chi^{2}$ distribution. This variance is then used to generate values from a normal distribution. DE genes are finally defined using levels compared to a threshold. The procedure used in [8,9] allows one to generate data with parameters derived from assumptions about real data. However, the percentage of the DE genes depends on a parameter that is difficult to control *a priori*. For some of the above methods, there is no distinction between the number of weakly expressed genes and those strongly expressed.

The model proposed in [10] provides synthetic data fairly close to the characteristics of true data. In this model, the level of expression of a gene is obtained by the superposition of several components. These components allow one to define the DE genes and the overall level of variability in the data. However, it lacks flexibility in choosing the number of DE genes and the number of genes over- and under-regulated. In this paper, we are interested in generating synthetic microarray data associated with two biological conditions. These conditions correspond to comparison of wild-type samples *versus* knock-out ones, treated samples *versus* non-treated ones, *etc*. For these situations, we use the generic terms of control *versus* test samples. Two condition biological data can be obtained using either one-color or two-color microarray technology leading to $log_{2}$ intensities or $log_{2}$ ratios, respectively. We assume that the same reference sample was used for $log_{2}$ ratio data.

This paper is organized as follows. In the next section, we present the model used and describe its parameter settings. In Section 3, we present commented results obtained using our model. Conclusions are drawn in Section 4.

## 2. Methods

We propose here a model that does not require knowledge of the physical laws governing gene expression to produce data with similar characteristics to the data commonly produced by current platforms. The user provides a subset of parameters that control the behavior of the data generated. We assume the following characteristics for microarray data:

1. There are usually more genes with low intensities than genes with high intensities. One possible explanation for this observation is a non-response of all genes to given biological conditions.
2. The $log_{2}$-intensities of microarray data usually vary between zero and 20. Expression values are the results of 16- or 20-bit float per pixel images, representing probes on arrays.
3. Under similar biological conditions, the expression level of a gene varies around an average value. Exceptionally different values will be due to technical problems.
4. The total number of DE genes depends on the biological conditions used. The number of over-regulated and under-regulated genes may be different.
5. The variability observed for weakly expressed genes is larger than that observed for highly expressed genes. This is mitigated with today’s scanners and tools. However, genes which are not really expressed still have non-zero values, and strongly expressed genes (saturated) receive a single value corresponding to the maximum level of quantification.

In the model described below, the expression levels are $log_{2}$ intensities. Ratios are derived from these values.

### 2.1. Model Used

Given *n* genes, $m_{1}$ and $m_{2}$ control and test samples, respectively, we used the following model:

(1) $$x_{i}=a_{i}+s_{i}+n_{i}+t_{i}$$

where $x_{i}$ is a vector of values generated for gene, *i*, (i=1,…,n). These values come from four sources. The values of vector $a_{i}=\left\{a_{ij},\;j=1,\ldots ,m_{1},m_{1}+1,\ldots ,m_{1}+m_{2}\right\},$ are expression levels for samples. The values of vector $s_{i}$ allows one to define DE genes. These values are zero for control samples and for genes that are not DE, while for DE genes, non-zero values are associated with test samples. The additive noise vector, $n_{i}$, has values independent of gene expression levels. Values associated with technical problems are cast in vector $t_{i}$, which has only a few non-zero components (samples) for some genes. The current implementation of our model does not include any technical problem terms, $t_{i}$, but we intend to take into account this component in the future.

To generate data using model (1) that satisfies the issues given above, we proceed as follows. The beta distribution is used to obtain *n* values varying between zero and one. Shape parameters ($shape_{1}$ and $shape_{2}$) of this distribution were chosen to produce more small values than higher ones (issue 1). The *n* values obtained are scaled to fit real data (issue 2), which vary between a lower bound (*lb*) and an upper bound (*ub*). The scaled values represent average expression levels for the *n* genes (issue 3). Optionally, real data can be used as seed at this step. We assume that intensities for each gene are uniformly distributed around its average level. The range of variation of values from this distribution is assumed to be dependent on the gene average level and is expressed as a percentage through a parameter, $\alpha =\lambda_{1}e^{-\lambda_{1}{\bar{z}}_{i}}$, where $\lambda_{1}$ is a parameter setting and ${\bar{z}}_{i}$ is the average level of gene, *i* (issue 5). This leads to vector $a_{i}$. Two parameters are used for generating vector $s_{i}$, the percentage of DE genes (pde) and a setting determining the number of up- and down-regulated genes (sym). The first $m_{1}$ values (control samples) of $s_{i}$ are set to zero. For gene *i*, an integer is randomly drawn from the set {1,…,100} and is compared to 100pde to decide whether the corresponding gene is DE. If the answer is yes, a second integer is drawn from the set {1,…,100} and is compared to 100sym to decide up and down assignment. $m_{2}$ normally distributed values are generated using a mean ($\mu_{de}$) and a standard deviation ($\sigma_{de}$). These values (or their opposite) form the second part (test samples) of vector $s_{i}$ for the up- (down-) regulated DE genes. Independent noise vector $n_{i}$ values are obtained using a normal distribution with zero mean and parameter ($\sigma_{n}$) for standard deviation. The details of this algorithm are given in Algorithm 1 and described in the following sections.

### 2.2. Model Parameters

#### 2.2.1. Data Size: *n*

This is the number of probes (genes) on the arrays. It is on the order of thousands, and the default setting is n=10,000.

#### 2.2.2. Number of Samples: $m_{1}$ and $m_{2}$

These parameters are the number of control and test samples, respectively. Typically, the minimum number of samples per condition should be three to allow the use of statistical tests. The maximum value of these parameters rarely exceeds 100 (samples per biological condition); the default setting is seven for both.

#### 2.2.3. Expression Level or Ratio: *Ratio*

This parameter option generates $log_{2}$ intensities or $log_{2}$ ratios data. With *ratio = 0* (default setting), $log_{2}$ intensities data will be generated; otherwise, $log_{2}$ ratios data are returned.

**Algorithm 1:** Steps of the micorarray data generation model.Generate *n* values from a beta distribution using $shape_{1}=2$ and $shape_{2}$ as shape parameters, we obtain values $z=\{z_{i},\;i=1,\ldots ,n\}$Transform values of z, such that they vary between settings *lb* and *ub*: $\bar{z}=lb+ub\times z$For each value of $\bar{z}$, generate $m_{1}+m_{2}+1$ uniformly distributed values: $a_{i}+s_{i}=\{U\left(\left(1-\alpha \right){\bar{z}}_{i},\left(1+\alpha \right){\bar{z}}_{i}\right)\}$, where $\alpha =\lambda_{1}e^{-\lambda_{1}{\bar{z}}_{i}}$ and $\lambda_{1}$ is a user setting.Then, the first $m_{1}+1$ values are used for a vector $r_{1}$, and the last $m_{2}$ values are used for a vector $r_{2}$.
(a)If gene *i* is differentially expressed (a sample ∈{1,…,100}<100pde)
Set $v_{1}=r_{1}$If probe *i* is up-regulated (a sample ∈{1,…,100}>100sym)
Generate $m_{2}$ normally distributed values and add them to $r_{2}$:$\mu_{de}=\mu_{de}^{min}+\left\{E\left(\lambda_{2}\right)\right\}$$v_{2}=r_{2}+\left\{N\left(\mu_{de},\sigma_{de}^{2}\right)\right\}$, where $\lambda_{2}$, $\mu_{de}^{min}$ and $\sigma_{de}^{2}$ are settingsElse
Generate $m_{2}$ normally distributed values and subtract them from $r_{2}$:$\mu_{de}=\mu_{de}^{min}+\left\{E\left(\lambda_{2}\right)\right\}$$v_{2}=r_{2}-\left\{N\left(\mu_{de},\sigma_{de}^{2}\right)\right\}$, where $\lambda_{2}$, $\mu_{de}^{min}$ and $\sigma_{de}^{2}$ are settingsSet: $y_{i}=a_{i}+s_{i}=\left(v_{1}\;v_{2}\right)$(b)Else
Set: $y_{i}=a_{i}+s_{i}=\left(r_{1}\;r_{2}\right)$Now, we have a noise-free data matrix $Y:=\{y_{i},i=1,\ldots ,n\}$. Do we need noisy data?
(a)If the noise standard deviation parameter is positive (setting $\sigma_{n}$)$N:=\{n_{i}=N\left(0,\sigma_{n}\right),\;i=1,\ldots ,n\}$
Add normally distributed values to data: $\bar{Y}=Y+N\}$,(b)Else
Do not use noise: $\bar{Y}=Y$Depending on user settings, $log_{2}$-intensities or $log_{2}$-ratios are returned
(a)If ratios are required
The first column of $\bar{Y}$ is used as reference: $X=\bar{Y}\left[,2:m_{1}+m_{2}+1\right]-\bar{Y}\left[,1\right]$(b)Else
$log_{2}$-intensities are returned: $X=\bar{Y}\left[,2:m_{1}+m_{2}+1\right]$


#### 2.2.4. Beta Distribution Shape Parameters: $Shape_{1}$ and $Shape_{2}$

To obtain more small values than high ones, we use a beta density distribution. Based on many histogram plots using various shape parameter values, we propose to set parameter $shape_{1}$ to two and allow the user to choose a value for the parameter $shape_{2}$ in the interval [4,8]. The default setting for $shape_{2}$ is four.

#### 2.2.5. $Log_{2}$ Intensities Variation Range: *lb* and *ub*

These parameters specify the $log_{2}$ intensities variation range. Quantification of microarray images is usually performed with 16- to 20-bit base, leading to $2^{16}$ to $2^{20}$ levels of gray. We suggest using values for parameters *lb* and *ub* from intervals [2,6] and [8,16], respectively. These values are typically observed for actual Affymetrix GeneChip${}^{©}$ array data for gene expression profiling. Default settings are lb=4 and ub=14. Observed minimum and maximum expression values will not exactly match the settings because of the variations used and possible additive noise.

#### 2.2.6. Percentage of DE Genes: *Pde*

This parameter controls the number of differentially expressed genes the user would like to have in the data set. Its values are taken from the interval [0,1]. A value pde=0.02 (default setting) means that 2% of the *n* probes in the data set should be DE.

#### 2.2.7. Number of Up- and Down-Regulated Genes: *Sym*

This parameter results in nearly the same number of up- and down-regulated probes when *sym = 0.5* (default setting). If the value of this parameter is less (greater) than 0.5, we will have more up- (down-) regulated probes in the data set.

#### 2.2.8. Gene Average Level Variation Range: $\lambda_{1}$ (*Lambda1*)

We assume that the values of each probe are uniformly distributed around an average value. An exponential distribution is used, $\alpha =\lambda_{1}e^{-\lambda_{1}{\bar{z}}_{i}}$, where $\lambda_{1}$ is a user setting for decreasing rate. Then parameter, *α*, controls the width of the uniform distribution and is expressed as a percentage of the average level. $\lambda_{1}$ allows a high variability in weakly expressed genes and, at the same time, a low variability for strongly expressed genes. We use the default value, $\lambda_{1}=0.13$, which allows an α≈10% for an average expression level equal to two and an α≈3.54% for an average expression level equal to 10. Increasing $\lambda_{1}$ will lead to more variability for weakly expressed genes and a small variability for strongly expressed genes. Small values for $\lambda_{1}$ (≈0.01) will lead to the same variability for all genes independently of their expression level.

#### 2.2.9. Fold Change Variation Parameters: $\lambda_{2}$, $\mu_{de}^{min}$ and $\sigma_{de}$ (*Lambda2, Muminde and Sdde*)

**Figure 1:**
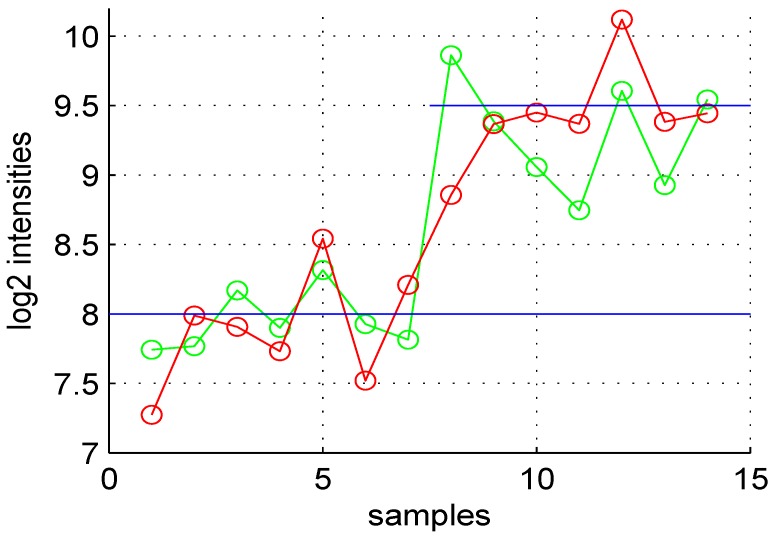
Variation of expression levels of a DE gene. The first seven points are for control samples, and the last seven points correspond to test samples. The average value of this gene in the control is eight and 9.5 for the test sample (blue horizontal lines). The green points correspond to noise free data, while the red are observed values for control and test samples.

For a gene, the fold change is a shift of average expression levels between test and control samples. Assuming that (control and test) sample values of a given gene are uniformly distributed around an average level, the shift comes from a superposition of additional values to the test samples, see Figure 1. We assume a normal distribution for the shift values $N(\mu_{de},\sigma_{de})$. However, the same mean $\mu_{de}$ is not used for all DE genes. Hence, we use a minimum value (setting $\mu_{de}^{min}$) and the exponential distribution to get $\mu_{de}=\mu_{de}^{min}+\left\{\lambda_{2}e^{-\lambda_{2}}\right\}$, where $\lambda_{2}$ is another setting. Default settings for these parameters are: $\mu_{de}^{min}=1.0$, $\lambda_{2}=2$ and $\sigma_{de}=0.5$. A higher $\lambda_{2}$ value will lead to a small number of genes having a shift greater than $\mu_{de}^{min}$; a small $\lambda_{2}$ value leads to the opposite situation. Parameters $\mu_{de}^{min}$ and $\sigma_{de}$ may be chosen using a one sample Student *t*-test analysis. The statistic of the shift value is $z_{s}=\frac{\sqrt{m_{2}}\mu_{de}^{min}}{\sigma_{de}}$. The critical value for a significance level of 0.05 for $z_{s}$ is 1.96. Hence, parameters may be chosen using the relation $\sigma_{de}\leq \frac{\sqrt{m_{2}}\mu_{de}^{min}}{1.96}\approx 0.51\sqrt{m_{2}}\mu_{de}^{min}$. The choice of additive noise standard deviation, $\sigma_{n}$ (see below), will modify this inequality.

#### 2.2.10. Additive Noise Standard Deviation: $\sigma_{n}$ (*Sdn*)

This parameter represents a normal distribution standard deviation for additive noise. The default value is $\sigma_{n}=0.4$. A zero value for this parameter will lead to noise-free data. Too high $\sigma_{n}$ values can lead to a number of DE genes different to those specified in *pde*.

#### 2.2.11. Computer Random Generator Seed: *Rseed*

This parameter is used for computer random number initialization. It will allow one to generate the same data at different times. The default value is 50.

## 3. Results and Discussion

To evaluate the performance of the proposed model, we performed simulations, in which we studied the influence of different parameter settings. Their default values are: n=10,000, $m_{1}=m_{2}=7$, lb=4, ub=14, $\lambda_{1}=0.13$, $\lambda_{2}=2$, $\mu_{de}^{min}=1.0$, $\sigma_{de}=0.5$, ratio=0, sdn=0.4, $shape_{2}=4$, pde=0.02, sym=0.5). We performed 100 independent simulations by changing the initialization of the generator through parameter, *rseed*. For each simulation, we performed a Student *t*-test and selected genes with a *p*-value less than 0.006. Using the DE information, selected genes were split into two: true and false DE genes. The *p*-value threshold, 0.006, leads to an expected error (false discovery rate) equal to (0.006×n)/(pde×n)=30% for default settings. When studying one parameter, the others are set to their default value.

### 3.1. Parameter Pde

**Figure 2:**
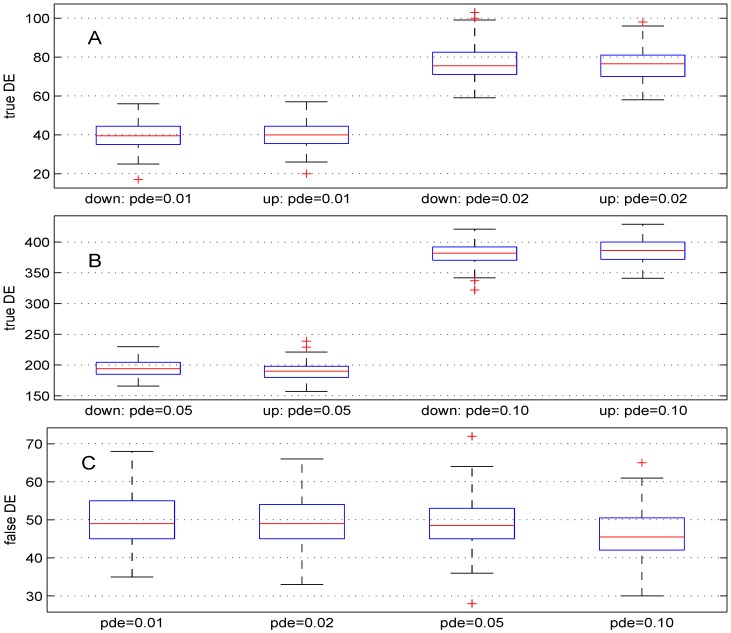
Boxplots of the number of down- and up-regulated genes (true DE, false DE) with four values of the parameter *pde*. 100 simulations were used for these results.

We used four different values (1%, 2%, 5% and 10%). For these values, the theoretical numbers of DE genes are, respectively, 100, 200, 500 and 1,000. The boxplots in Figure 2 show the results obtained. The median numbers of true down- (up-) regulated genes are 39.5 (40), 75.5 (76.5), 194 (190) and 382 (386.5) for the above values of parameter *pde*, respectively. In comparison with the expected number of DE genes, the recovery powers of the Student *t*-test are 79.5%, 76%, 76.8% and 76.8%. Better power results can be obtained for this test by using smaller value for parameter $\sigma_{n}$. Panel C of Figure 2 shows the number of false DE genes obtained using the four values for parameter *pde*. The median numbers of the false DE genes are 49, 49, 48.5 and 45.5 for the above values of parameter *pde*, respectively.

### 3.2. Parameter *Sym*

**Figure 3:**
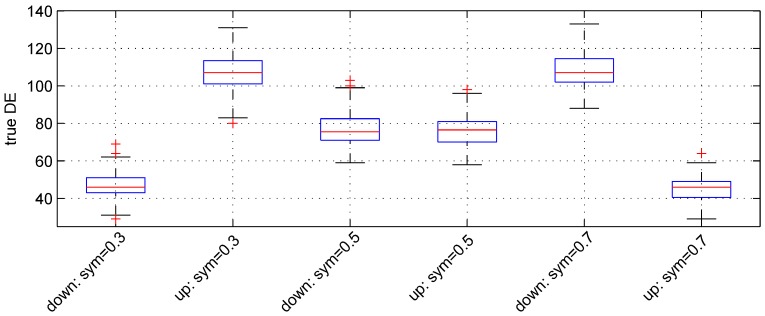
Boxplots of the number of down- and up-regulated genes (true DE) with three values of the parameter *sym*. 100 simulations were used for these results.

We used the following three values: 0.3, 0.5 and 0.7. For these values, the expected numbers for pairs of down- and up-regulated genes are, respectively, (60,140), (100,100) and (140,60). Figure 3 shows the boxplot of results obtained. The median numbers of true down- and up-regulated genes observed are (46, 107), (75.5, 76.5) and (107, 46) for the above values of parameter sym, respectively. Hence, the recovery powers of the Student *t*-test are 76.5%, 76% and 76.5%.

The median number of false DE genes is 49 for the three values of parameter *sym*.

### 3.3. Parameter $\sigma_{n}$

**Figure 4:**
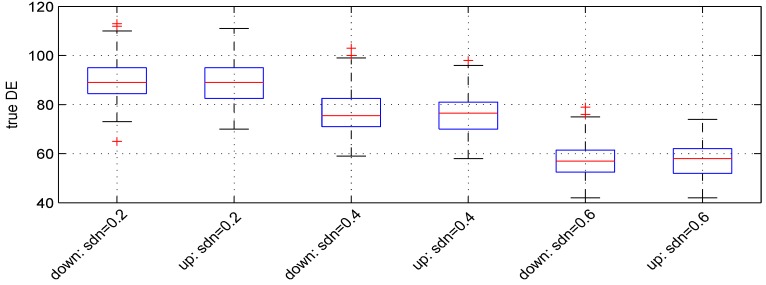
Boxplots of the number of down- and up-regulated genes (true DE) with three values of the parameter $\sigma_{n}$. 100 simulations were used for these results.

We used three values: 0.2, 0.4 and 0.6. Figure 4 shows the boxplots of the results obtained. The median numbers of down- and up-regulated genes observed are (89, 89), (75.5, 76.5) and (57, 58), leading to detection powers of 89%, 76% and 57.5%, respectively. The *t*-test detection power decreases when $\sigma_{n}$ increases.

The median numbers of false DE genes are 54, 49 and 49 for the 3 values of parameter $\sigma_{n}$, respectively.

### 3.4. Parameters $\mu_{de}^{min}$, $\sigma_{de}$, $\lambda_{1}$, $\lambda_{2}$ and $Shape_{2}$

We performed simulations to examine the influence of these parameters. For each parameter, 100 simulations were used, and the results obtained are summarized in Table 1. Increasing the parameter $\mu_{de}^{min}$ setting introduces more change for the DE genes, while its decrease leads to the opposite effect. Parameter $\sigma_{de}$ acts as noise. The effect of the modification of some parameters is investigated further in the MA plot representations described in the following paragraph.

**Table 1:** Number of down- and up-regulated detected genes using the Student *t*-test and various parameter settings.

Parameters	(down, up)	power
$\mu_{de}^{min}=2$	(100, 100)	100%
$\mu_{de}^{min}=0.5$	(41, 40)	40.5%
$\sigma_{de}=0.2$	(90.5, 90)	90%
$\sigma_{de}=0.4$	(82, 83)	82.5%
$\sigma_{de}=0.6$	(69, 69.5)	69%
$\lambda_{1}=0.1,\sigma_{n}=0.4$	(76, 6)	76%
$\lambda_{1}=0.01,\sigma_{n}=0.4$	(81, 81)	81%
$\lambda_{1}=0.1,\sigma_{n}=0.2$	(89, 88)	88.5%
$\lambda_{1}=0.01,\;\sigma_{n}=0.2$	(92, 92)	92%
$\lambda_{2}=4,$	(66, 66)	66%
$\lambda_{2}=0.5$	(92, 91)	91.5%
$shape_{2}=4$	(75.5, 76.5)	76%
$shape_{2}=6$	(75, 76)	76.5%
$shape_{2}=8$	(76, 76.5)	76%

### 3.5. Volcano and MA Plots

**Figure 5:**
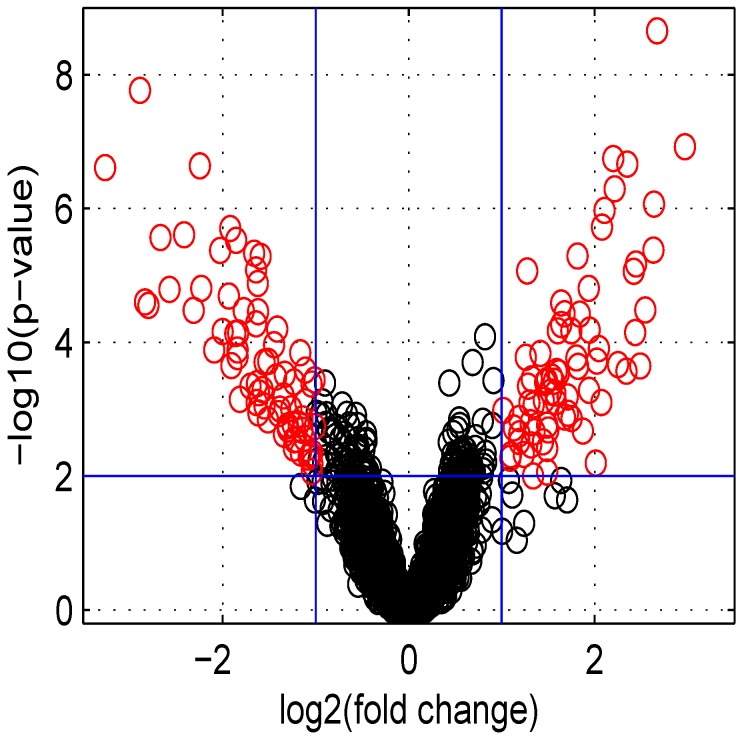
Volcano plot of data obtained.

**Figure 6:**
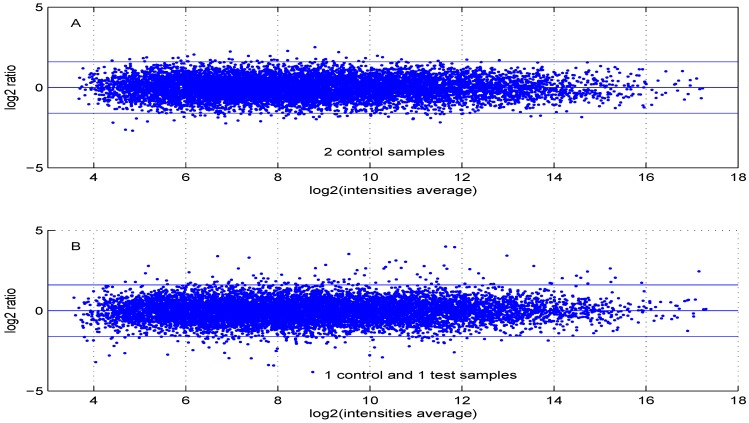
MA plot using (**A**) two control samples or (**B**) one control and one test sample data.

Using default settings, we performed one simulation. Then we computed the Student *t*-test *p*-value and fold change for all genes. These values were used in the volcano plot of Figure 5. Red circles represent genes having a *p*-value less than 0.01 and a fold change greater than two or less than 0.5. Intensity measurements of two samples can be used to create two new variables: $M=I_{x_{2}}-I_{x_{1}}$ and $A=0.5(I_{x_{2}}+I_{x_{1}})$, where $I_{x_{2}}$ and $I_{x_{1}}$ are $log_{2}$ intensities of samples $x_{2}$ and $x_{1}$, respectively. A value, one (-1) for M means that the corresponding gene is up- (down-) regulated two-fold. A plot of *M* ($log_{2}$ ratio) *versus A* ($log_{2}$ intensities average) is denoted “MA plot" [11]. The MA plots in Figure 6 are obtained using either two control samples (panel A) or one control and one test sample (panel B).

**Figure 7:**
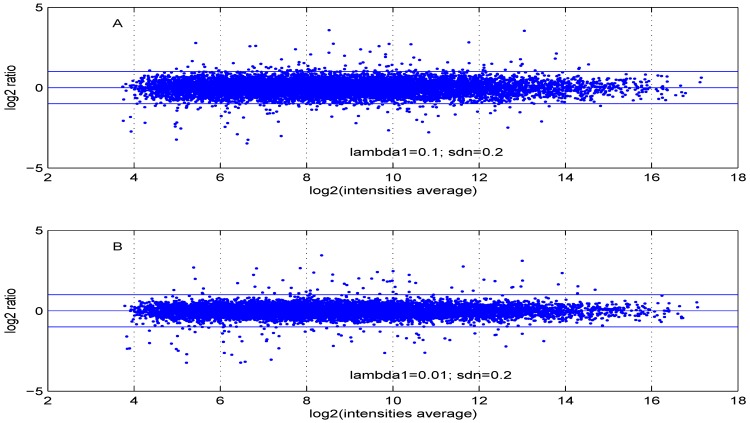
MA plot with one control and one test sample, using (**A**) $\lambda_{1}=0.1,\sigma_{n}=0.2$ or (**B**) $\lambda_{1}=0.01,\sigma_{n}=0.2$.

**Figure 8:**
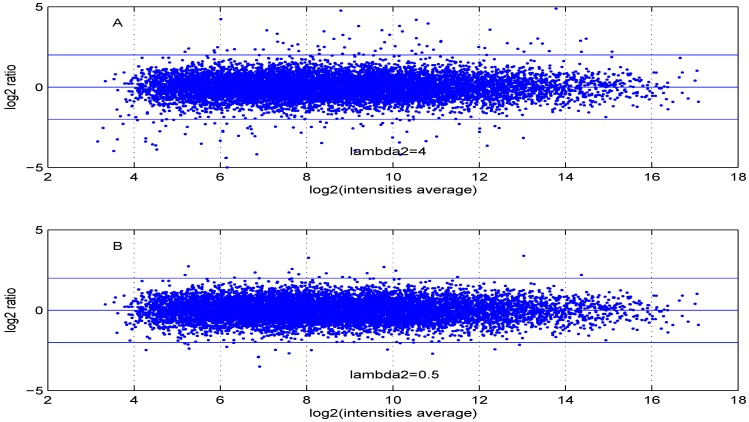
MA plot with one control and one test sample, using (**A**) $\lambda_{2}=4$ or (**B**) $\lambda_{2}=0.5$.

**Figure 9:**
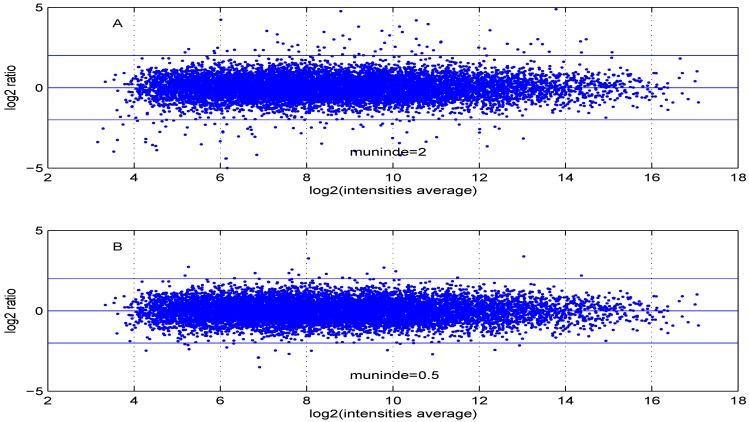
MA plot with one control and one test sample, using (**A**) $\mu_{de}^{min}=2$ or (**B**) $\mu_{de}^{min}=0.5$.

**Figure 10:**
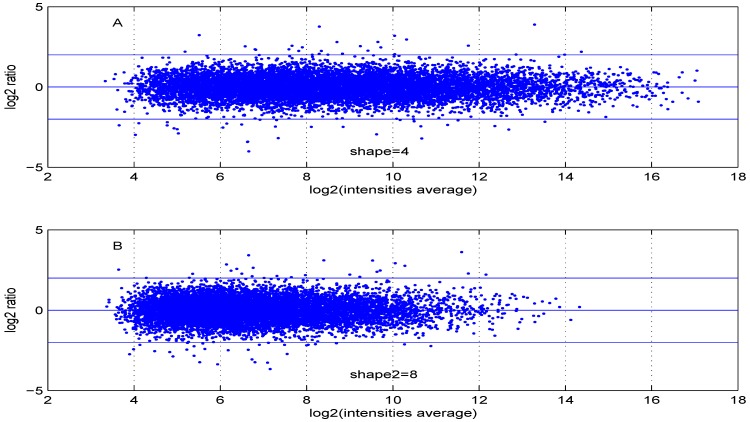
MA plot with one control and one test sample, using (**A**) shape2=4 or (**B**) shape2=8.

Additional MA plots in Figure 7, Figure 8, Figure 9 and Figure 10 showing the effect of some parameter settings. A small value for $\lambda_{1}$ leads to a less dense cloud of points. A larger change is observed for higher values of $\lambda_{2}$ than for smaller ones. The same applies to parameter, $\sigma_{de}$. Increasing the parameter $shape_{2}$ value leads to a decrease of the dynamic range of the data.

### 3.6. Discussion

The choice of some setting parameters for the proposed model is easy and can be dictated by the experimental design. This applies to *n*, $m_{1}$, $m_{2}$ and *ratio*. Parameters *lb* and *ub* and *sym* and *pde* concern the dynamic range of variation of the data to generate and define the DE genes. The intervals indicated for *lb* and *ub* are those observed for data from common platforms. Parameters, $shape_{2}$, lb and ub, have no effect if real microarray data are used as a seed. The number of DE genes (*pde*) and the proportion of under- and over-regulated genes (*sym*) is at the discretion of the user. The parameter, *rseed*, allows one to produce the same data at different times using the same computer. This parameter is also useful for generating test data for different analysis algorithms. Settings, $\lambda_{1}$, $\lambda_{2}$, $\mu_{de}^{min}$, $\sigma_{de}$ and $\sigma_{n}$, control the global behavior of the data generated. More precisely, $\lambda_{1}$ allows one to make gene changes dependent on the average level of expression. $\lambda_{2}$ introduces variation in the expression changes for DE genes. These changes are defined by $\mu_{de}^{min}$ and $\sigma_{de}$. Parameter, $\sigma_{de}$, also acts as noise. The parameter, $\sigma_{n}$, allows one to perturb the data generated. The example of Figure 4 shows that the data obtained can differ from those expected for large values of $\sigma_{n}$. The signal to noise ratio (defined as the ratio of standard deviations) for default settings is 6.25. This rises to 4.16 for $\sigma_{n}=0.6$.

An interesting microarray study was reported in [12]. In that study, the same RNA samples were processed by many laboratories using three leading microarray platforms: Affymetrix (five labs), two-color cDNA (three labs) and two-color oligonucleotides (two labs). The results presented show a good agreement across-platforms in contrast to some results previously reported in the literature, see, for instance, references cited in [12]. The microarray data generated for the study described in [12] are available from the Gene Expression Omnibus [13] under accession number GSE2521. These data can be used as seed for our model, which can then be integrated into user friendly data analysis software, such as Partek Genomics Suite, GeneSpring GX, *etc.*, for demonstration and/or teaching purposes.

### 3.7. R Code

For immediate use of the proposed model, we provide an R code function *madsim.R* (MicroArray Data Simulation Model), which is deposed as a package on the Comprehensible R Archive Network (CRAN) server for download [14]. The outputs of this function are the data generated (*xdata*) and the indexes of DE genes (*xid*). Real data can be used as seed for each gene. An example of such data is available in the data folder of the package. Further explanations are available from the package’s help function.

## 4. Conclusions

We proposed in this paper a simulation model of microarray data. This model is very flexible and allows one to generate data with similar characteristics to the data commonly produced by current platforms. We showed a commented example of its possible use. We considered the case of data from two biological conditions. This model can be extended to multiple biological conditions in different ways: (a) modify to take into account additional biological conditions and several levels for the parameters, $\mu_{de}$ and $\sigma_{de}$, and (b) use as is, then place data side by side.
